# Insights into the Function of Aquaporins in Gastrointestinal Fluid Absorption and Secretion in Health and Disease

**DOI:** 10.3390/cells12172170

**Published:** 2023-08-29

**Authors:** Giuseppe Calamita, Christine Delporte

**Affiliations:** 1 Department of Biosciences, Biotechnologies and Environment, University of Bari Aldo Moro, 70125 Bari, Italy; giuseppe.calamita@uniba.it; 2 Laboratory of Pathophysiological and Nutritional Biochemistry, Faculty of Medicine, Université Libre de Bruxelles, 1070 Brussels, Belgium

**Keywords:** aquaporin channels, exocrine glands, endocrine glands, membrane transport

## Abstract

Aquaporins (AQPs), transmembrane proteins permeable to water, are involved in gastrointestinal secretion. The secretory products of the glands are delivered either to some organ cavities for exocrine glands or to the bloodstream for endocrine glands. The main secretory glands being part of the gastrointestinal system are salivary glands, gastric glands, duodenal Brunner’s gland, liver, bile ducts, gallbladder, intestinal goblet cells, exocrine and endocrine pancreas. Due to their expression in gastrointestinal exocrine and endocrine glands, AQPs fulfill important roles in the secretion of various fluids involved in food handling. This review summarizes the contribution of AQPs in physiological and pathophysiological stages related to gastrointestinal secretion.

## 1. Introduction

Aquaporins (AQPs) represent a family of proteins expressed in all living organisms that contain six transmembrane domains with both N- and C-terminal ends located intracellularly [1]. AQPs have a tetrameric organization conferring their functionality [1,2]. The mammalian family of AQPs is made of thirteen members (AQP0 to AQP12) [1]. Some AQPs are mostly permeable to water (AQP0, AQP1, AQP2, AQP4, AQP5, AQP6, AQP8), while others are also permeable to glycerol (AQP3, AQP7, AQP9, AQP10, AQP11) [1,3] or urea (AQP3, AQP6, AQP7, AQP9, AQP10) [4]. The permeability of one AQP still remains to be specified (AQP12) [4]. Some AQPs also ensure the transport of gas, such as carbon dioxide (AQP0, AQP1, AQP4, AQP5, AQP6, AQP9), nitric oxide (AQP1, AQP4), ammonia (AQP1, AQP3, AQP6, AQP7, AQP8, AQP9) and oxygen (AQP1) [4]. In addition, some AQPs can facilitate the movement of hydrogen peroxide (peroxiporins: AQP1, AQP3, AQP5, AQP8, AQP11), some ions (AQP0, AQP1, AQP6), silicon (AQP3, AQP7 AQP9, AQP10), antimonite and arsenite (AQP7, AQP9) [4].

AQPs are distributed throughout the entire body and fulfill a wide range of physiological functions [1,5]. AQPs are expressed in exocrine and endocrine glands to ensure glandular secretion [6]. This review summarizes the involvement of AQPs in the physiology of glandular secretion ensured by the main exocrine and endocrine glands distributed along the gastrointestinal tract and being involved in food handling. Hence, a particular focus is put on the role of AQPs in glandular secretion ensured by salivary glands, stomach glands, duodenal Brunner’s glands, intestinal Goblet cells, bile-secreting glands and pancreas. Moreover, for each gland, we address the role of relevant AQPs in association with diseases and, when relevant, the new therapeutic options and perspectives to modulate AQPs function in some of the secretory glands.

## 2. Salivary Glands

Saliva secretion is ensured by major and minor salivary glands [7,8]. Major salivary glands, present in pairs, include parotid glands located in front of and below each ear, submandibular glands located below the jaw, and sublingual glands located under the tongue. Minor salivary glands line the upper aerodigestive tract mucosa and most of the oral mucosa. The salivary glands are organized into lobes made of lobules. Each lobule consists of clusters of acinar cells, forming the acini, and interconnected ductal cells, forming the ducts. The acini and some ducts are surrounded by myoepithelial cells facilitating saliva secretion upon contraction [9]. The serous, mucous and seromucous acini (made respectively of serous, mucous or a mixture of both serous and mucous acinar cells) contribute to the secretion of a watery and mucus-rich secretion [9]. The ductal system is made of intralobular (intercalated and striated), interlobular and interlobar (excretory) ducts. 

### 2.1. Role of AQPs in Saliva Secretion

**Figure 1:**
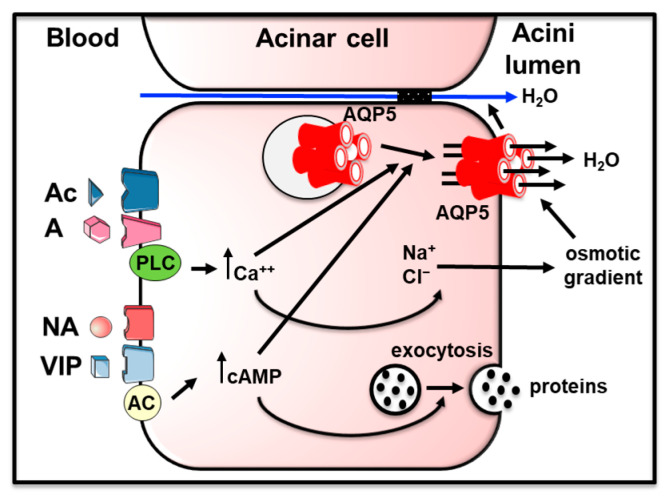
Role of AQP5 in saliva production in acinar cells. Upon neuronal excitation, acetylcholine, adrenaline, noradrenaline and vasoactive intestinal peptide bind to their receptor and induce either the activation of phospholipase C or adenylyl cyclase resulting in an increase in the intracellular calcium or cyclic adenosine monophosphate (cAMP) concentration. This leads to the trafficking of intracellular vesicles containing AQP5 to the apical plasma membrane and the secretion of sodium and chloride. The latter creates an osmotic gradient allowing the passage of water to the acini lumen. AQP5 is also suggested to play the role of osmosensor involved in the control of paracellular flow as well. A: adrenalin; Ac: acetylcholine; AC: adenylyl cyclase; Ca^++^: calcium ions; cAMP: cyclic adenosine monophosphate; Cl^−^: chloride ions; NA: noradrenaline; Na^+^: sodium ions; PLC: phospholipase C; VIP: vasoactive intestinal peptide. Figure was adapted from [6], reproduced with permission from SNCSC, the Licensed Material is not part of the governing OA license but has been reproduced with permission).

Saliva formation involves both acinar and ductal cells. At first, acini produce an isosmotic fluid rich in NaCl and water. Then, ducts ensure the reuptake of some of the NaCl and the bicarbonate secretion. These combined processes lead to the secretion of hypotonic saliva into the oral cavity [10,11]. AQP5, located at the apical membrane of acinar cells, ensures water movement to the lumen of the acini following sodium and chloride secretion creating an osmotic gradient, playing a major role in saliva secretion [12,13]. Indeed, *Aqp5* knockout mice presented a 60% decrease in pilocarpine-stimulated saliva secretion, as well as more viscous and hypertonic saliva [12,13]. Furthermore, acinar cells from parotid and submandibular glands from these mice displayed decreased water permeability (65% and 77%, respectively) [13]. From these studies and others, it was inferred that AQP5 is responsible for transmembrane water movement across the apical plasma membrane of acinar cells [10,11,14,15]. It was suggested that molecular mechanism involving AQP5 allows transcellular and paracellular water movement in a collective manner [16,17]. Under physiological conditions, muscarinic stimulation of acinar cells leads to intracellular calcium increase triggering AQP5 translocation to the plasma membrane [18,19,20]. Concomitantly, serous acini from all human salivary glands [21,22] and from rat [22,23,24,25] and mouse [18,26,27] submandibular and parotid glands mainly express AQP5 at their apical plasma membrane. Figure 1 summarizes the role of AQP5 in saliva formation in acinar cells. AQP5 expression was also reported in rat and mouse ductal cells [18,25,28,29]. However, physiologically, this localization is difficult to explain since ductal cells are water impermeable [30]. Rats with an AQP5 point mutation displayed reduced AQP5 expression and saliva secretion [31]. However, to the best of our knowledge, no human AQP5 mutation has so far been linked to salivary gland dysfunction.

In addition to AQP5, other AQPs are present in salivary glands [32,33]. Indeed, AQP1 is expressed in mouse [34] and human [21,22,35,36,37] endothelial and myoepithelial cells and in rat endothelial cells [29,38,39,40,41]. In human salivary glands, AQP3 was localized at the basolateral membrane of serous and mucous acinar cells but not in ductal cells [21,36,37], and *Aqp4*, *Aqp6* and *Aqp7* mRNAs but not their proteins have also been detected [21,37]. In rats, the expression of both AQP3 and AQP4 remains a subject of debate [28,29,42,43]. In mice, AQP3, AQP4 and AQP8 are expressed at the basolateral membrane of acinar and ductal cells [34]. Rat parotid glands expressed AQP6 in secretory granule membrane [44]. In mouse salivary glands, AQP7 was detected in the endothelial cells [27]. AQP8 is expressed in salivary gland myoepithelial cells [45,46,47]. In mouse salivary glands, AQP9 distribution remains to be fully assessed [26,27,33] and AQP11 was detected in ductal cells [26,27]. Moreover, different profiles of AQPs expression have been documented during the development of salivary glands in mice, rats and humans [29,48,49,50,51]. The employment of *Aqp1*, *Aqp4* and *Aqp8* knockout mice has ruled out their physiological involvement in saliva secretion [12,52,53]. 

### 2.2. Role of AQPs in Diseases Affecting Salivary Gland Function

Abnormal AQP5 expression and/or distribution has been associated with some pathophysiological conditions affecting salivary glands including Sjögren’s syndrome, head and neck cancer treated with ionizing radiation therapy, and diabetes.

#### 2.2.1. Sjögren’s Syndrome

Sjögren’s syndrome is an autoimmune disease characterized by lymphocytic infiltration of exocrine glands, in particular salivary and lacrimal glands, often leading to xerostomia (dry mouth) and keratoconjunctivitis sicca [54]. Due to the frequent xerostomia observed in patients with Sjögren’s syndrome, it was hypothesized that altered AQP5 expression and/or localization may account for this observation [22]. Impaired AQP5 localization was detected [22,55] or not [56,57,58] in the salivary glands of patients with Sjögren’s syndrome. The use of distinct patient subsets and/or antibodies may account for these apparent divergent data. In several mouse models of Sjögren’s syndrome, modified AQP5 distribution has indisputably been documented [59,60,61,62,63,64]. Several parameters may account for the altered AQP5 localization [65] such as the presence of inflammatory infiltrates [61], cytokines [66,67,68,69], autoantibodies against muscarinic M3 receptors [70,71], alteration of the machinery regulating intracellular calcium levels [65,72,73,74]. Very recently, AQP5 interacting protein partners have been suggested to regulate AQP5 trafficking in salivary glands from patients with Sjögren’s syndrome [75,76]. Indeed, both ezrin and prolactin-inducible protein, two newly identified protein partners of AQP5 in salivary glands, presented altered expression and/or localization that may account for abnormal AQP5 localization [75,76]. Both prolactin-inducible protein and ezrin interacted with the C-terminus end of human AQP5 [75,76]. Anti-AQP5 antibodies, recently discovered in blood samples of patients with Sjögren’s syndrome, may also take part in salivary gland dysfunction [77]. So far, patients- and methodologies-related variabilities may account for the heterogeneity in the detection of anti-AQP5 antibodies among patients suffering from Sjögren’s syndrome [78] and their association with decreased unstimulated saliva flow [77,79]. Therefore, further studies will be necessary to assess whether anti-AQP5 antibodies lead to salivary gland dysfunction. Rituximab, a B-cell depleting agent, reverted the decreased AQP5 expression detected in the salivary gland and the reduced saliva flow [80]. The beneficial effect of Rituximab on unstimulated saliva flow rate was confirmed in a larger cohort of patients with Sjögren’s syndrome [81], but it remains to assess if this effect may be due to restored AQP5 expression. Overall, abnormal AQP5 localization in salivary glands from patients with Sjögren’s syndrome resulting from multifactorial events may occur in subsets of patients and participate in salivary gland dysfunction. 

Modified expression of other AQPs has been found in the salivary glands of patients with Sjögren’s syndrome. Indeed, lower AQP1 [36] and AQP4 [82] expression in myoepithelial cells have been documented. Treatment of a patient suffering from Sjögren’s syndrome with Rituximab reverted the decreased AQP1 expression detected in the salivary gland [80]. However, no association has been found between AQP1 autoantibodies detected in patients with Sjögren’s syndrome [78,83] and reduced saliva flow rate [83]. Besides, saliva secretion was not modified in *Aqp1* and *Aqp4* knockout mice [12,52]. Overall, these data suggest no involvement of AQP1 and AQP4 in saliva secretion. 

#### 2.2.2. Other Diseases

Reduced or loss of AQP5 expression [84,85] and/or altered AQP5 translocation [86] may participate to xerostomia in patients with head and neck cancer treated with ionizing radiation therapy. In addition, decreased AQP5 expression and saliva secretion were also detected following irradiation of the neck area in mice and rats [87,88,89,90,91] and could be reverted in rats by administration of pilocarpine, a muscarinic receptor agonist [92]. 

In diabetes, high glucose concentration impaired [93] or not [94] AQP5 distribution and expression [95]. These divergent data may result from the use of distinct mouse species, experimental conditions, and analytical methods. 

### 2.3. New Therapeutic Options to Modulate AQPs in Diseases Affecting Salivary Glands 

To alleviate xerostomia, AQPs have been targeted or modulated by pharmaceutical approaches and have also been used in gene therapy. In vitro, DNA demethylation agents increased AQP5 expression in salivary gland cell lines [68,96]. In vivo, the use of cevimeline, a pharmacological muscarinic agonist agent, restored AQP5 trafficking in salivary glands of different rat models characterized by impaired AQP5 localization [97,98,99]. In addition, the cystic fibrosis transmembrane regulator (CFTR) corrector and potentiator restored AQP5 expression and salivary gland function in a mouse model of Sjögren’s syndrome [100]. Very recently, in a mouse model of Sjögren’s syndrome, anticeramide treatment using myriocin was reported to attenuate inflammation, increase AQP5 expression, and to re-establish salivary gland function [101]. Gene therapy using recombinant adenoviral or adeno-associated viral vectors coding for AQP1 (AdhAQP1) were capable to restore saliva flow to irradiated salivary glands of animals and human with Sjögren’s syndrome [102,103,104,105,106,107], as well as to resolve inflammation [108]. In the clinic, patients with radiotherapy for head and neck cancer or with Sjögren’s syndrome represent ideal target populations for AQP1 gene therapy. However, the advance of AQP1 gene therapy relies on the development of new viral vectors promoting more efficient and persistent gene expression, as well as less immune reactivity. The substitution of the endogenous AQP1 gene promoter with the cytomegalovirus (CMV) promoter using CRISPR-CAS9 gene editing increased AQP1 expression in vitro [109]. Overall, if limitations related to gene therapy can be overcome later on, gene therapy may be clinically beneficial to patients suffering from xerostomia consequent to head and neck cancer treated with ionizing radiation therapy or Sjögren’s syndrome. 

## 3. Gastric Glands

Mammalian gastric glands are sitting in invaginations formed by the epithelium of the gastric mucosa (gastric pits). According to their localization, they are classified into cardiac (in the cardia of the stomach), fundic (in the body and fundus of the stomach) and pyloric glands (in the antrum of the pylorus connecting the stomach to the duodenum). Gastric glands are composed of a variety of cells including several types of mucous cells producing mucous, progenitor cells replenishing the gastric epithelium, parietal cells secreting gastric acid and bicarbonate ions, chief cells releasing pepsinogen, D cells secreting somatostatin, G cells secreting gastrin, X cells secreting ghrelin, enterochromaffin cells releasing serotonin and enterochromaffin-like cells releasing histamine [110]. 

### 3.1. Function of AQPs in Gastric Secretion

The stomach expresses many AQPs. *Aqp1*, *Aqp3*, *Aqp4*, *Aqp5*, *Aqp7*, *Aqp8*, *Aqp10* and *Aqp11* mRNAs have been detected in the fundus, while *Aqp1*, *Aqp2*, *Aqp 3*, *Aqp 5*, *Aqp 7*, *Aqp 11* mRNAs have been detected in the antrum of the pylorus [111,112,113]. However, the presence of only a few AQPs has been confirmed at the protein level, including AQP3, AQP4, and AQP5 as described in more detail hereafter.

Upon histamine stimulation, AQP4 located at the basolateral plasma membrane of chief and parietal cells [43,114,115,116,117] undergo phosphorylation and internalization in the vesicle-recycling compartment to escape the degradative pathway [118]. Internalized AQP4 can recycle back to the plasma membrane following histamine wash out. Therefore, AQP4 can dynamically be regulated by endocytosis and recycling. It was proposed that the simultaneous decrease in histamine response and localization of AQP4 at the cell surface account for the down-regulation of gastric secretion [118]. However, the hypothesized role of AQP4 in water movement accompanying gastric secretion has been ruled out using *Aqp4* knockout mice [119], thereby suggesting a compensatory role of other AQPs in such a process. Additional studies are necessary to assess if AQP4 may still play a role in the maintenance of the volume of gastric cells. The function of AQP5, located at the apical and lateral membranes of pyloric glands [120], remains to be determined in the stomach. 

### 3.2. Pathophysiological Involvement of AQPs in Gastric Diseases

Quite a few AQPs have been involved in pathogenic mechanisms of diseases affecting the stomach, in particular, gastric cancer and chronic gastritis [113,121,122,123,124,125,126,127,128]. 

AQP3 and AQP5 ensure significant functions in gastric cancer [129]. They induce the invasion and metastasis of gastric cancer cells by regulating epithelial-mesenchymal transition [123,130]. Diminished levels of miR-877, miR874, and miR370 increased AQP3 expression and epithelial-mesenchymal transition [131,132,133]. The levels of long noncoding RNA LINC00659, acting as a molecular sponge of miR-370, were upregulated in gastric cancers and associated with tumor stage, metastasis and poorer prognosis [133]. LINC00659 knockdown suppressed the proliferation, metastasis and epithelial-mesenchymal transition in gastric cancer cells in vitro [133]. Mechanistically LINC00659 competes with miR-370 to increase AQP3 expression in gastric cancer [133]. Recent work reported the identification and validation of AQP5 as a potentially specific marker of gastric cancer stem cells, as well as the role of AQP5 in the self-renewal and tumorigenesis of gastric cancer stem cells by complementing the effect of leucine-rich repeat-containing G protein-coupled receptor 5 (LGR5) [134]. Mechanistically, AQP5 regulated the autophagy and stemness of gastric cancer stem cells by recruiting E3 ligase Tripartite Motif Family Like 1 (TRIM21) to the key autophagy protein Unc-51 Like Autophagy Activating Kinase 1 (ULK1) and inducing the ubiquitination of ULK1 [134]. In Epstein-Barr virus (EBV)-associated gastric cancer, the EBV-encoded protein latent membrane protein 2A (LMP2A) could down-regulate AQP3 by inhibiting the activation of Mechanistic Target Of Rapamycin Kinase (mTOR) signaling pathway and further inhibit autophagy and migration of gastric cancer cells [135]. Furthermore, AQP3 increased the expression of Eukaryotic Translation Initiation Factor 4E Binding Protein 1 (EIF4ABP1 also named 4E-BP1; a target of mTOR) and its phosphorylation by activating Mitogen-Activated Protein Kinase 1 (MAPK1; also named Extracellular Signal-Regulated Kinase (ERK)) signaling pathway, which induced autophagy and promoted cell proliferation in gastric cancer cells [135]. *Helicobacter pylori* infection, representing the major cause of chronic gastritis, can promote AQP3 and AQP5 expressions which correlate with the progression of gastric carcinoma [136,137]. *Helicobacter pylori* induced both AQP3 and AQP5 expression by activating downstream HIF-1a or ERK1/2, MEK, respectively [136,137]. The expression of some AQPs in gastric cancer may be used as predictive prognostic gastric cancer biomarkers considering their association with overall patient survival [127,138]. 

Low *Aqp4* expression may represent a good biomarker for spasmolytic polypeptide-expressing metaplasia (SPEM) (a regenerative lesion of the gastric mucosa that can evolve towards cancer). The lowered gastrin levels in SPEM [139,140] may account for the decreased *Aqp4* expression as gastrin can regulate *Aqp4* expression [141]. 

### 3.3. New Therapeutic Options to Modulate AQPs in Diseases Affecting Stomach

Considering their role in gastric cancers, AQPs may represent additional molecular therapeutic targets [142]. Moreover, targeting specific miRNA and long noncoding RNA involved in the regulation of AQPs may also provide additional therapeutic options in the future.

## 4. Duodenal Brunner’s Gland and Intestinal Goblet Cells

Mammalian duodenal Brunner’s glands lie in the submucosal layer of the proximal duodenum and are innervated by cholinergic vagal and polypeptidergic nerves releasing acetylcholine and vasoactive intestinal peptide (VIP), respectively. In most mammalian species, they are found in decreasing abundance from the pylorus-duodenum junction to the biliary/pancreatic duct [143]. The glands, made of clusters of serous cells and branching ducts, secrete a fluid rich in mucin glycoproteins containing a limited amount of bicarbonate and a variety of peptides (such as trypsin inhibitors, antimicrobial peptides, and growth factors). Intestinal goblet cells are scattered in the epithelia of the small intestine and secrete a fluid rich in mucins [144]. The role of goblet cells relies on the secretion of fluid serving as an important barrier to prevent pathogens to invade the intestinal mucosa and cause inflammation, and in the delivery of luminal antigens to antigen-presenting cells to initiate adaptative immune response [145]. 

### 4.1. Role of AQPs in Fluid Secretion of Duodenal Brunner’s Gland and Intestinal Goblet Cells 

So far, the roles of AQPs in duodenal Brunner’s gland and intestinal goblet cells have been poorly studied. In Brunner’s gland cells, the expression of AQP5 has been detected at the apical, lateral and secretory granule membranes [120] and that of AQP1 at the level of the apical and lateral membranes [146]. VIP stimulated the secretion of bicarbonate and proteins and increased the secretion rate of rat Brunner’s gland [147] by mechanisms involving a cAMP-dependent signaling cascade inducing translocation of AQP5, but not of AQP1, from secretory granules to the apical plasma membrane [143,146]. Once located at the apical plasma membrane, AQP5 is anticipated to facilitate water movement and thereof contribute to increased fluid secretion. The co-localization and co-trafficking of CFTR with AQP5 support the existence of a parallel pathway for electrolyte secretion and osmotic water movement [143]. Additional studies using *Aqp5* knockout mice will be valuable to corroborate the involvement of AQP5 in Brunner’s gland secretion. Concerning the expression of AQPs in intestinal goblet cells, only *Aqp9* mRNA has been documented in a subset of these cells [148]. Overall, further experiments are necessary to obtain more precise data concerning the expression and function of AQPs in both these duodenal and intestinal secretory cells. 

### 4.2. Role of AQPs in Diseases Affecting the Duodenal Brunner’s Glands and Intestinal Goblet Cells

Celiac disease and cystic fibrosis have been characterized by decreased AQP5 expression in Brunner’s glands which may contribute to the altered duodenal secretion occurring in these diseases [143]. However, the participation of AQP5 in the pathogenesis of celiac disease and cystic fibrosis requires further investigation. To the best of our knowledge, no data are available concerning the role of AQPs in diseases affecting intestinal goblet cells. However, if AQPs are involved in mucin secretion, their dysregulation is anticipated to impair intestinal homeostasis and participate in diseases associated with goblet cells dysfunction, such as colitis and infections.

## 5. Glands Ensuring Bile Secretion

Bile is a complex biologic fluid rich in water (95%) which contains bile acids, cholesterol, phospholipids, bile pigments, proteins, metabolites, hormones, and inorganic ions [149]. Bile ensures the excretion of cholesterol (in the form of unesterified cholesterol or as bile acids, the latter assisting the emulsification and absorption of lipid compounds in the duodenum) and the elimination of various toxins and drugs. In healthy conditions, adult humans produce each day between 0.8 to 1.0 L of hepatic bile daily at a rate of 30–40 mL per hour [150]. Bile fluid formation begins at the canalicular (apical) membrane of hepatocytes. It involves the secretion of ions and osmotically-active compounds-mainly through the bile salt transporter (e.g., the bile salt export pump (BSEP)) and the organic anion transporter (e.g., the multidrug resistance-associated protein 2, (MRP2)) creating the osmotic force that drives the parallel water flow [150]. The composition of human canalicular bile is then modified by the epithelial cells lining the ductal lumen. Bile is stored and concentrated in the gallbladder (cystic bile), and released into the duodenum during fat digestion [151,152]. While bile water is mostly recovered in the proximal portion of the small intestine [153] bile salts are reabsorbed in the distal ileum from where they return to the liver via the enterohepatic circulation [154,155]. Bile acid-independent bile flow also exists and results from an active secretion of *osmotically active* inorganic electrolytes and organic anions. Bile formation results from continuous ductal/ductural secretion and canalicular bile flow [150].

In mammals, the epithelial cells lining the hepatobiliary tree express a variety of AQPs with distinct subcellular localizations (Table 1). As in the blood vessels of other body districts, the endothelial cells of the hepatobiliary system express AQP1 [41].

### 5.1. Role of AQPs in Glands Ensuring Bile Secretion

#### 5.1.1. Liver

Rodent and human hepatocytes express high levels of AQP8 and AQP9 [156,157,158,160,161,169]. Murine hepatocytes [170] and immortalized human hepatocyte cell line Huh7 also express AQP11 [156]. AQP3 and AQP7 have also been detected in the human liver [156]. The redundancy in AQP expression may be due to their distinctive subcellular localization and molecular selectivity [159,171]. In hepatocytes, AQP8, AQP9 and AQP11 fulfill significant functions, whereas the roles of AQP3 and AQP7 in hepatocytes remain unclear.

**Figure 2:**
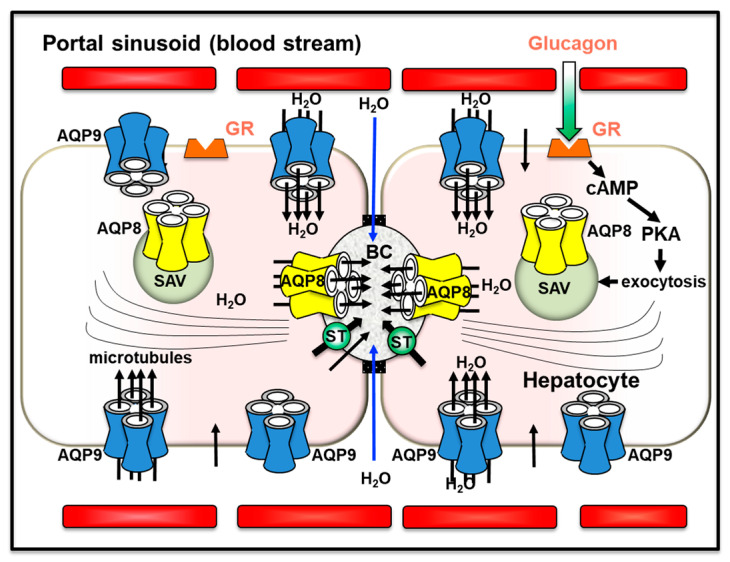
Role of AQP8 and AQP9 in canalicular bile formation and secretion in hepatocytes. AQP8 mediates the osmotically-driven transport of water into the bile canaliculus across the canalicular membrane, whereas AQP9 ensures the diffusion of water from the sinusoidal blood into the hepatocytes. An AQP-independent movement of water also occurs through the paracellular route passing across the tight junctions to enter the bile canaliculus (blue arrows). Glucagon triggers the microtubule-dependent canalicular targeting of AQP8-containing subapical vesicles. AQP8, present in the mitochondria and the smooth endoplasmic reticulum, may ensure additional roles beyond facilitating the canalicular secretion of bile water. AQP9 ensures the main pathway for the import of lipolytic glycerol from sinusoidal blood (see Table 1). cAMP: cyclic adenosine monophosphate; BC: bile canaliculus; GR: glucagon receptor; PKA: protein kinase A; SAV: subapical vesicles; ST: salt transporters. Figure was adapted from [6], reproduced with permission from SNCSC, the Licensed Material is not part of the governing OA license but has been reproduced with permission).

In hepatocytes, AQP8 shows multiple subcellular localizations that range from the canalicular membrane and subapical vesicles to organelles such as the mitochondria and the smooth endoplasmic reticulum [157,169]. Hepatocyte AQP8 has been suggested to mediate the secretion of canalicular bile water [172], preserve the cytoplasm osmolarity during glycogen synthesis or degradation [169], facilitate the ammonia movement in mitochondrial ammonium detoxification and ureagenesis [173,174,175,176] and mediate the efflux of hydrogen peroxide out of mitochondria during the oxidative stress [177,178]. AQP8 may exert a role in the cholesterol biosynthesis modulated by the sterol regulatory element-binding protein (SREBP) [179,180,181]. Based on its peroxiporin activity, AQP8 has recently been implicated in the differential regulation of metabolic signaling by α1- and β-adrenoceptors (ARs) leading to the induction of Ca^2+^ ion mobilization [182]. Extensive work has been carried out in investigating the role of AQP8 in canalicular bile secretion [159]. Choleretic agonists such as dibutyryl cAMP or glucagon induced the translocation of subapical vesicles incorporating AQP8 to the canalicular plasma membrane through a phosphatidylinositol-3-kinase (PI3K)-dependent microtubule-associated pathway [183]. This was accompanied by an increase in the water permeability of the canalicular plasma membrane with consequent osmotic movement of water into the bile canaliculus [172,184,185] (Figure 2). Glucagon also increased the AQP8 expression by decreasing its degradation via a mechanism involving cAMP-PKA and PI3K signal pathways in rat primary hepatocytes [186].

The pleiotropic functional relevance of AQP9 results from its permeability to various neutral solutes (i.e., glycerol and other polyols, H_2_O_2_, urea, carbamides, nucleosides, monocarboxylates, purines, pyrimidines and metalloid arsenic) in addition to water [187]. In rodent hepatocytes, AQP9 is localized at the sinusoidal domain of the basolateral plasma membrane [161] and ensures the principal entry pathway for blood glycerol into hepatocytes during fasting [188,189,190,191]. Imported glycerol is rapidly converted into glycerol-3-phosphate (G3P) acting as a major substrate for gluconeogenesis during early starvation. AQP9 has also relevance in lipid homeostasis since G3P is needed for triacylglycerols (TAGs) synthesis [192]. Hepatocyte AQP9 has been involved in rodent bile formation and in extruding catabolic urea by facilitating the entry of water and the exit of urea from and to portal blood, respectively [193,194]. In rodents, the negative regulation of *Aqp9* transcription by insulin [195] may account for enhanced hepatic AQP9 expression in insulin resistance [196,197]. The functional relevance of AQP9 in metabolic homeostasis and energy balance was also revealed by the diminished liver glycerol permeability and the enhanced concentrations of plasma glycerol and TAGs detected in AQP9-depleted knockout mice [193,198]. Reduction in AQP9 levels and liver glycerol permeability were seen in hepatocytes of murine models of obesity and subjects with obesity with type 2 diabetes [199,200]. The hepatic expression of AQP9 is also controlled by leptin [192,201]. However, the modulation exerted by these hormones on AQP9 appears to be different between humans and rodents [197]. Gender-related dimorphism of liver AQP9 expression is seen both in rodents and humans in line with the distinctions between the two sexes in handling glycerol for metabolic purposes [201]. In rat hepatoma cell lines, AQP9 played a role in the lipid-lowering activity of silybin, a nutraceutical phytocompound, through modulation of the autophagic process and lipid droplet composition [202]. Liver AQP9 seems also to have immune relevance considering its involvement in the early acute phase of the inflammatory response triggered by TLR4 ligands in mice [203]. Furthermore, the inhibition of AQP9 with the selective and potent blocker RG100204 abolished the LPS-induced increase in NO and O_2_^−^ levels in FaO hepatoma cells [204].

Mouse liver AQP11 was involved in rough endoplasmic reticulum homeostasis and liver regeneration through a mechanism that remains unclear [156,170]. The recent functional identification of AQP11 as a peroxiporin suggests its potential implication in the regulation of intracellular H_2_O_2_ balance to avoid ER stress [205]. Additional studies will be valuable to determine the exact role of this AQP in the liver.

#### 5.1.2. Bile Ducts

**Figure 3:**
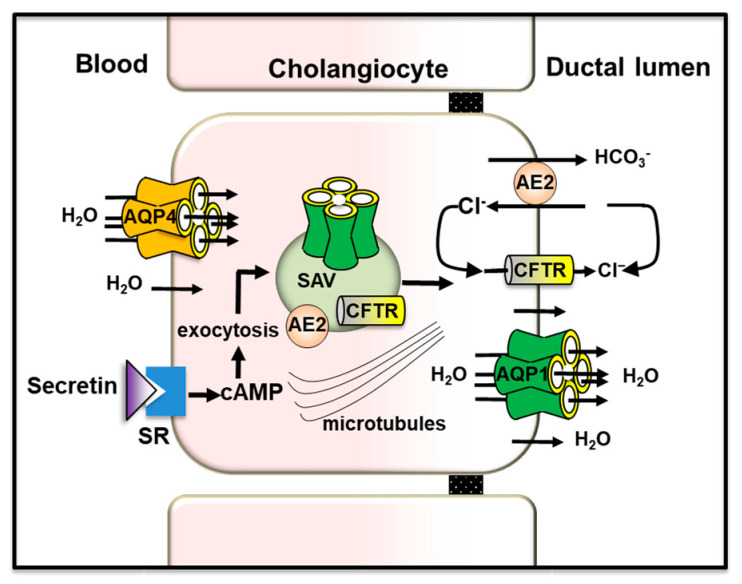
Role of AQP1 and AQP4 in bile secretion in intrahepatic duct cholangiocytes. Secretin, through cAMP, induces the microtubule-dependent apical targeting and translocation of subapical vesicles containing AQP1, CFTR (Cl^−^ channels) and AE2 (Cl^−^/HCO_3_^−^ exchangers) to the apical plasma membrane. The exit of Cl^−^ via CFTR drives the extrusion of HCO_3_^−^ into the ductal lumen through AE2. The presence of HCO_3_^−^ and Cl^−^ ions generate an osmotic gradient allowing the movement of water from blood plasma (via AQP4 located at the basolateral membrane) to the biliary lumen (via AQP1 located at the apical membrane). AE2: anion exchanges isoform 2; CFTR: cystic fibrosis transmembrane conductance regulator; SAV: subapical vesicles; SR: secretin receptor. Figure was adapted from [6], reproduced with permission from SNCSC, the Licensed Material is not part of the governing OA license but has been reproduced with permission).

In cholangiocytes, secretin induces bile secretion through a cascade of events including the activation of the cAMP signaling pathway [206], a Cl^−^ efflux via CFTR driving the extrusion of HCO_3_^−^ into the lumen via apical AE2 (chloride/bicarbonate exchanger) and an osmotically-driven water movement through AQP1 located at the apical membrane [206]. In human and rodent cholangiocytes, AQP1 mediates the apical secretion of water during both basal- and secretin-induced ductal bile formation [41,207,208]. AQP1 is also found in subapical membrane vesicles [162], co-expressed with AE2 and CFTR [209], undergoing exocytic insertion into the apical membrane of cholangiocytes upon secretin stimulation [162,209]. In the new notion of functional bile secretory unit, AQP4 and AQP1 located at the basolateral and apical plasma membrane of cholangiocytes [162,210] are allowing water movement to ensure the maintenance of the relative iso-osmolar status of the cell during ductal bile formation. This novel concept is in line with the physical association that exists between the cholangiocyte basolateral membrane and the peribiliary vascular plexus that surrounds the bile ducts and from which bile water derives [159,211] (Figure 3).

However, the water permeability of cholangiocytes isolated from AQP1-depleted knockout mice was not diminished [212]. Compensatory upregulation of other mouse cholangiocytes AQPs (i.e., AQP8) has been invoked to explain this observation [213,214]. Intrahepatic bile ducts not only secrete but also absorb water as demonstrated in isolated rodent intrahepatic bile duct units [151]. It was hypothesized that osmotic absorption of water is triggered by the active uptake of sodium-coupled glucose and bile salts through the sodium/glucose transporter 1 (SGLT1) and apical sodium-dependent bile acid transporter (ASBT), respectively [206]. Somatostatin, gastrin and insulin may decrease ductal bile secretion by lowering the intracellular levels of cAMP and the subsequent translocation of AQP1, CFTR and AE2 induced by secretin. This mechanism may explain why somatostatin reduces ductal secretion while stimulating the net absorption of ductal water.

#### 5.1.3. Gallbladder

**Figure 4:**
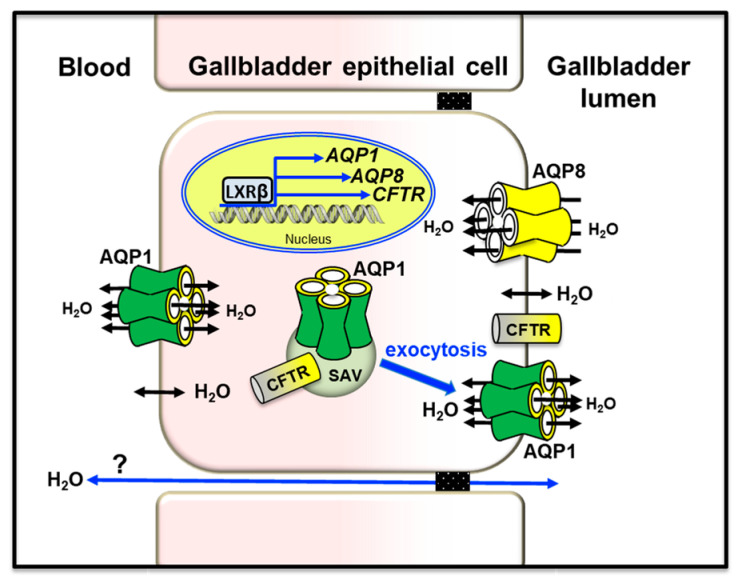
Role of AQP1 and AQP8 in gallbladder bile absorption/secretion. Apical AQP8 and AQP1 promote the osmotic absorption and secretion of water into/from the gallbladder lumen, respectively. Basolateral AQP1 allows the entry/extrusion of water into/out of the epithelial cells. AQP-independent movement of water likely occurs through the tight junctions (paracellular route; blue arrow). The nuclear receptor LXRβ has been suggested to regulate bile volume and the expression of AQP1 and AQP8 together with that of CFTR in the gallbladder of male mice [218]. Thus, the flow of water through the gallbladder epithelium would occur by means of multiple mechanisms involving AQPs and CFTR. CFTR: cystic fibrosis transmembrane conductance regulator. LXRβ: liver X receptor β. SAV: subapical vesicle.

The mammalian gallbladder stores bile produced through hepatobiliary secretion and plays key roles in lipid digestion and maintenance of metabolic homeostasis. The movement of water across the gallbladder epithelium is led by osmotic forces generated from active salt absorption and secretion [159,215]. Both human and murine gallbladder epithelial cells express AQP1 and AQP8. AQP1 is present both at the apical and at the basolateral plasma membrane of the epithelial cells that line the neck of the organ [163,216]. AQP1 is also localized at the corpus portion in subapical vesicles and plasma membrane [164]. In the murine gallbladder, AQP1 was slightly upregulated by leptin [217]. AQP8 is present at the plasma membrane and, to a lower extent, in intracellular vesicles of the gallbladder epithelium of various species [41,157]. In gallbladder cholangiocytes, AQP1, AQP8, and CFTR were up-regulated by the liver X receptor β (LXRβ), an oxysterol-activated transcription factor highly present in the gallbladder epithelium [218]. A molecular partnership between CFTR and AQPs has also been found in mouse Sertoli cells [219]. In mouse gallbladder epithelium, AQP1 accounted for high water permeability [165]. The osmotic water permeability was independent of cAMP and osmotic gradient size and direction. As in bile duct cholangiocytes, subapical AQP1 is translocated to the apical membrane to ensure water movement across the gallbladder epithelium. Based on its subcellular pattern of localization, gallbladder AQP8 is likely involved in the absorption of water and, to a lesser extent, in the secretion of water into the lumen [157] (Figure 4). However, the exact relevance of AQP1 and AQP8 in gallbladder function remains a debated argument due to contradictory reported results. Indeed, similar bile salt concentrations were found from gallbladders of wild-type and AQP1-ablated mice, with no apparent functional substitution of AQP1 by AQP8 [165]. However, this observation was not supported by the temporal association observed between diminished gallbladder concentrating function and decreased AQP1 or AQP8 levels [164], and the effects leptin replacement in leptin-deleted mice altering the gallbladder volume acting on the AQP-mediated absorption/secretion of water [220]. Additional studies will be worth to clarify the question.

### 5.2. Role of AQPs in Diseases Affecting the Glands Secreting Bile

Various diseases that affect the hepatobiliary tree have been linked to perturbed bile fluid secretion and resulting cholestasis [159,221]. Dysregulated hepatobiliary AQPs and altered bile secretion have been observed in various experimental models of cholestasis. Studies with cellular and murine models of gallstone disease suggest an association between altered AQP expression/localization in cholangiocytes and gallbladder concentrating function [159].

#### 5.2.1. Liver and Bile Ducts

Dysregulated expression of AQP8 at the canalicular side of hepatocytes contributes to the development of cholestasis in several experimental models of cholestasis including extrahepatic obstructive cholestasis [222], estrogen-induced cholestasis [223] and sepsis-induced cholestasis [224]. The association between downregulation of canalicular AQP8 and decreased canalicular osmotic water permeability suggests relevance for AQP8 in cholestasis [207,223]. The conjoint occurrence of reduced solute transport and impaired water permeability may account for cholestasis [225]. Interestingly, adenoviral transfer of the human AQP1 gene to rat liver improved bile flow in estrogen-induced cholestasis with potential therapeutic implications for cholestatic diseases [226]. New considerations have been made regarding the occurrence of cholestasis and its progression [227,228] taking into account that a reduction in the paracellular or transcellular canalicular water flow had no significant effect on bile acid excretion [229]. Basolateral AQP9, facilitating the movement of water from the sinusoidal blood into the hepatocyte, was downregulated at a post-transcriptional level in a rodent model of extrahepatic cholestasis [194]. Hepatic cystogenesis accompanied by altered expression and subcellular distribution of AQP1 (together with CFTR and AE2) was seen in a rat model of autosomal recessive polycystic kidney disease. Liver cysts likely resulted from the increased fluid accumulation triggered by the overexpression and ectopic localization of AQP1, CFTR, and AE2 in cystic cholangiocytes [230]. Disruption of the *Aqp11* gene in mice leads to intracellular vacuolization of periportal hepatocytes and a severe form of polycystic kidney disease (PKD) with uremic death before weaning due to renal failure [170]. Since the life span of *Aqp11*^−/−^ mice was limited by kidney disease, the liver phenotype could be premature. Polycystic livers are expectable in *Aqp11* knockout mice since cysts are often seen in the biliary epithelia of patients and mice with PKD [231]. Further studies are needed to assess whether the PKD provoked by the depletion of *Aqp11* in mice triggers the same liver cysts induced by the autosomal recessive form of PKD, a well-known form of PKD caused by homologous congenital polycystic kidney.

#### 5.2.2. Gallbladder

Gallstone disease (cholecystolithiasis) is characterized by a high prevalence (10–15% in adulthood) and cost in Western countries [232,233]. The majority of gallstones are cholesterol gallstones, while the minority are pigment stones that contain less than 30% cholesterol [234]. The prevalence of gallstones augments with age, while being associated with multiple risk factors [232,235]. Cholesterol gallstone disease results from altered cholesterol homeostasis inducing crystallization of bile cholesterol and reduced gallbladder contractility [236]. Cholesterol gallstone disease is currently considered as the hepatobiliary expression of the metabolic syndrome, being frequently associated with obesity, type 2 diabetes, dyslipidemia, and hyperinsulinemia. Reduced expression of the gallbladder AQP1 and AQP8 was associated with a decrement of the gallbladder concentrating ability in mice fed a lithogenic diet [164]. Dysregulated *Aqp1* and *Aqp4* mRNA levels were found in the gallbladder of leptin-deficient obese [Lep(ob)] mice undergoing leptin replacement [217]. Besides showing the characteristic obesity, Lep(ob) mice show enhanced gallbladder volumes and diminished gallbladder contractility, the latter reflecting gallbladder stasis [220]. However, in humans, no significant relationship has been found between AQP1 and AQP8 expression in the gallbladder epithelium and the occurrence of gallbladder stones [163]. A recent study has reported that sex-specific expression and localization of hepatobiliary AQPs, associated with reduced biliary water movement, may account for lower cholesterol gallstone prevalence in female thyroid hormone (TH) deficient mice [237]. Additional experiments will be valuable to better understand the function of AQPs in gallstone disease.

### 5.3. New Therapeutic Options Using AQPs in Diseases Affecting Bile Secretion

Downregulated canalicular expression of BSEP and MRP2 and AQP8 plays a key role in the development of hepatocellular cholestasis [150,238]. There are still no studies aimed at modulating the expression of endogenous AQP to counteract clinical conditions with reduced bile secretion. However, compelling experimental evidence indicates that hepatic AQP gene transfer may improve hepatocellular cholestasis [239]. Recent studies using rodent models of hepatocellular cholestasis showed that the hepatic transfer of an adenoviral vector encoding for human AQP1 (AdhAQP1) improves bile secretion and re-establishes high serum levels of bile salts [226,240,241,242]. Interestingly, the AdhAQP1-transduced hepatocytes showed that the heterologous expression of hAQP1 at the canalicular membrane led to an increase in the osmotic membrane water permeability with an induction of the transport activities of BSEP and MRP2 by their redistribution in canalicular lipid rafts likely through the interaction with the cholesterol-binding protein caveolin-1.

## 6. Pancreas

### 6.1. Exocrine Pancreas

Histologically, the exocrine pancreas represents a major part of the pancreas (±90%). The glandular structure of the exocrine pancreas is very similar to that of salivary glands but presents few particularities. Indeed, the exocrine pancreas is made of only serous acinar cells, contains centroacinar cells (extension of intercalated ducts into each acinus) and a main collecting duct ensures pancreatic fluid drainage.

The pancreatic fluid, controlled by neurotransmitters (i.e., acetylcholine, cholecystokinin and secretin), neutralizes the stomach acid and ensures food digestion [243].

#### 6.1.1. Role of AQPs in Exocrine Pancreatic Secretion

The distribution of AQPs in the exocrine pancreas varies according to species. In the human exocrine pancreas, despite the detection of several *Aqps* mRNA (i.e., *Aqp1*, *Aqp3*, *Aqp4*, *Aqp5*, *Aqp8* and *Aqp12*), the protein expression of only a few of these AQPs has been confirmed (i.e., AQP1, AQP5 and AQP8) [244,245]. AQP1 was located in endothelial cells, centroacinar cells (apical membrane), intercalated ductal cells [244] and pancreatic zymogen granules [246,247]. AQP5 and AQP8 were, respectively, located in the apical membrane of both intercalated ductal cells and acinar cells, respectively [244].

In rat exocrine pancreas, *Aqp1*, *Aqp4*, *Aqp5*, *Aqp8* but no *Aqp12* mRNA have been detected [244,245,248]. AQP1 was localized to intralobular (apical and basolateral membrane) and interlobular ductal cells (apical and basolateral membranes and caveolae and vesicle-like structures) [249,250], acinar zymogen granules [246] and endothelial cells [248]. AQP5 was detected in centroacinar and intercalated ductal cells (apical membrane) [251]. AQP8 was located at the apical plasma membrane of acinar cells [245].

The murine exocrine pancreas expresses AQP1 and AQP5 in interlobular ductal cells (apical membrane), AQP5 in intercalated and intralobular ductal cells (apical membrane) [251], and AQP12 in acinar cells (intracellularly) [252].

Pancreatic juice output originates from the secretion of a small isotonic fluid by acinar cells. In acinar cells, water movement is ensured through AQP8 and AQP1 (located at the apical membrane). The content of the initial pancreatic fluid is then modified by the ductal cells which secrete ions and ensure the majority of the water movement to the ductal lumen via AQP1 (located at the apical and basolateral membranes) and AQP5 (located at the apical membrane) [10,251,253]. In rats, AQP8 is responsible for most water permeability (90%) in acinar cells [248]. However, Aqp8 knockout mice show normal exocrine pancreatic function, possibly due to the major contribution of ductal cells to transcellular water transport [53]. In rats, AQP1 contributes to basal and GTP-mediated vesicle water movement and swelling of pancreatic acinar zymogen granules [246,247]. In rat interlobular ductal cells, AQP1 is responsible for most of the pancreatic juice secretion induced by secretin [250]. However, both *Aqp1* and *Aqp5* knockout mice show no modification in exocrine pancreatic function [244], suggesting their low contribution to pancreatic fluid secretion and/or the presence of compensatory mechanisms. Therefore, the use of double *Aqp1* and *Aqp5* knockout mice are anticipated to precise the overall contribution of these AQPs to the pancreatic juice formation. A study using *Aqp12* knockout mice has suggested AQP12 may participate in the mechanisms that control the exocrine pancreatic secretory function in response to following rapid and intense stimulation [254]. Very recently, the contribution of AQP12 to pancreatic juice secretion has also been revealed in patients presenting a nonsense mutation in the *Aqp12B* gene, one of the two homologous *Aqp12* genes (*Aqp12A* and *Aqp12B*) [255]. Overall, additional studies are still necessary to evaluate the specific contribution of each AQP to pancreatic fluid secretion.

#### 6.1.2. Role of AQPs in Diseases Affecting Exocrine Pancreatic Secretion

Some AQPs have been incriminated in the pathogenesis of pancreatitis, cystic fibrosis and cancer [129,256,257,258]. Indeed, AQP1 expression was decreased in a rat model of acute necrotizing pancreatitis [259] and in a mouse model of pancreatic exocrine insufficiency [260]. However, the role of AQP1 in pancreatitis remains to be further explored. Beyond affecting the pancreatic function, pancreatitis can affect the lung and colon characterized by impaired AQPs expression [261,262,263]. AQP5 expression was decreased in a mouse model of autoimmune pancreatitis [100]. In that model, the CFTR corrector C18 and the CFTR potentiator VX770 rescued CFTR expression, corrected AQP5 expression and pancreatic fluid secretion and alleviated tissue inflammation [100]. However, the effects of these molecules on the AQP1 expression remain to be assessed. Nevertheless, CFTR correctors may represent strong candidates for the treatment of pancreatitis. Considering gene therapeutic approaches have been considered in pancreatic diseases [264], it may also be interesting to evaluate the effects of viral vectors encoding AQP1 or AQP5 [102,103,107,265].

*CFTR*, located in ductal cells, participates in pancreatic fluid secretion [266]. In cystic fibrosis, a genetic disease resulting from autosomal mutations in the *CFTR* gene, pancreatic juice secretion is altered [267]. In guinea-pig and mouse models of cystic fibrosis, the underlying mechanisms causing decreased AQP1 expression and reduced pancreatic fluid secretion remain to be elucidated [268,269].

In various cancers, AQPs have been involved in cell proliferation, migration, adhesion, invasion, metastasis, drug resistance, angiogenesis and epithelial-mesenchymal transition [270,271,272,273,274,275,276,277]. AQPs have been implicated in the pathogenesis of pancreatic ductal adenocarcinoma (PDAC), the most prevalent, aggressive and lethal pancreatic cancer [256,278]. The role of AQPs in cell proliferation, migration, and apoptosis have been studied using PDAC cell lines [256,278,279]. In addition, AQP3 and AQP5 have been implicated not only in cell migration but also in cell-cell-adhesion by modulation of cell biomechanical properties in a PDAC cell line [280]. Using a panel of PDAC cell lines, *miR-874* was shown to regulate *Aqp3* mRNA expression which promoted cell proliferation and survival (via increased expression and activity of mTOR and downstream targets) [281]. However, the role of AQPs in PDAC tissues has been less investigated. The expression of *Aqp1*, *Aqp3*, *Aqp4*, *Aqp5* and *Aqp8* mRNAs were detected in PDAC [244]. Moreover, *Aqp1* and *Aqp3* mRNA were upregulated in PDAC as compared to healthy pancreatic tissue [282]. The expression of AQP1 and AQP3 protein were increased in PDAC, predictive markers of poor prognosis, but associated with patients’ survival [258,281,282]. However, a study reported decreased *Aqp3* and *Aqp8* mRNA expression in PDAC [283]. These discrepant data may result from distinct patient cohort characteristics and analytic methodologies. Regarding AQP5 protein in PDAC, its localization was altered, and its expression was increased (more in moderately differentiated than in poorly differentiated PDAC) and correlated with tumor differentiation status and aggressiveness [258]. Further studies will be warranted to better understand the involvement of AQPs in PDAC, their usefulness as predictive or prognostic markers and their contribution to chemo-resistance.

### 6.2. Endocrine Pancreas

As opposed to the exocrine pancreas, the endocrine pancreas only accounts for a minor portion of the entire pancreatic tissue (±10%). Despite its minor representation, the endocrine pancreas fulfills important endocrinological functions by ensuring the secretion of two major metabolic hormones regulating glucose metabolism such as insulin and glucagon as well as other functionally relevant peptide hormones. The endocrine pancreas consists of islets of Langerhans scattered among the pancreatic tissue. Islets of Langerhans contain insulin-producing ß-cells surrounded by glucagon-producing *α*-cells, somatostatin-producing *δ*-cells, and pancreatic polypeptide-producing PP cells [284]. The primary role of the B-cells is to release insulin during the postprandial state to decrease glycemia [285,286]. Insulin secretion by B-cells results from the succession of several events. The first event consists of the entrance of glucose through the glucose transporter type 2 (GLUT2). Then the metabolization of glucose induces an increase in the intracellular ATP content which inhibits ATP-sensitive K^+^ channels that leads to membrane depolarization, and subsequent opening of voltage-dependent Ca^2+^ channels. The resulting intracellular calcium elevation triggers the exocytosis of insulin-containing granules into the bloodstream [285]. Furthermore, glucose induces b-cell swelling [287] which causes the activation of volume-regulated anion channel (VRAC) with consequent cell membrane depolarization and in turn the activation of voltage-dependent Ca^2+^ channels. The latter allows calcium entry which triggers insulin secretion [288,289]. 

#### 6.2.1. Role of AQPs in Endocrine Pancreatic Secretion

Very recently, the presence of *Aqp7* mRNA has been reported in a-, b- and d-cells from human islets of Langerhans [290]. Rat β-cells express AQP7 [291,292,293] and mouse β-cells express AQP5, AQP7, AQP8 [292] and AQP12 [294]. Further studies are still required to obtain a comprehensive map of AQPs distribution in human and rodent islets of Langerhans.

While data have ruled out the involvement of AQP12 in insulin secretion [294], several studies have pointed out the role of AQP7 in the regulation of intracellular glycerol content, insulin production and secretion in b-cells. Indeed, *Aqp7* knockout mice were characterized by reduced β-cell size and mass, insulin content and cAMP-driven glycerol release [293,295]. Furthermore, the mice displayed higher basal and glucose-stimulated insulin secretion rates, glycerol and TAG contents and glycerol kinase activity [293]. The mice’s genetic background are likely influencing the phenotype of the *Aqp7* knockout mice, i.e., the presence of hyperinsulinemia [293,295] combined with the presence [295] or absence [293] of hyperglycemia, or the presence of normoglycemia with undetermined insulin levels [296].

**Figure 5:**
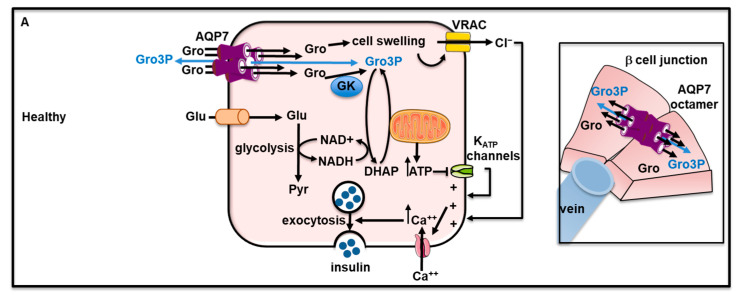

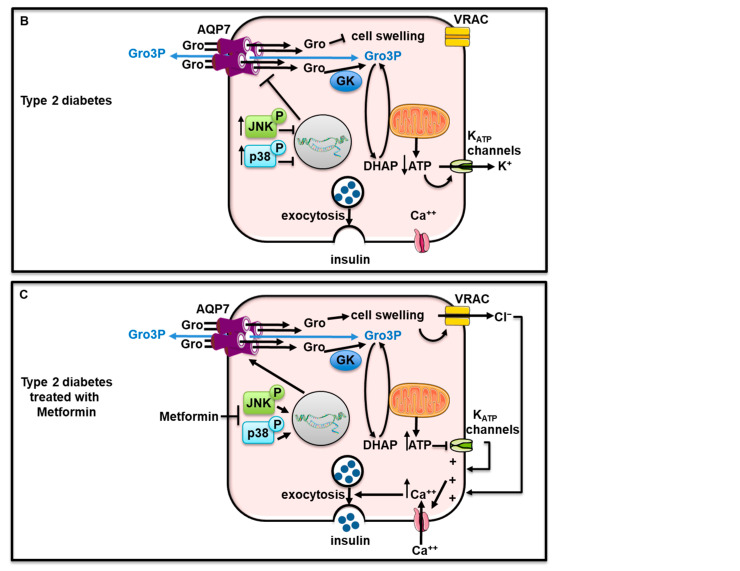
Role of AQP7 in β-cell. (**A**) In healthy β-cells, glycerol entry via AQP7 leads to successive cell swelling, VRAC activation, membrane depolarization, calcium entry, and insulin secretion. In addition, it is hypothesized that AQP7 may act as a junction protein by participating in β-cell rosette formation and intercellular movement and distribution of glycerol as well as other metabolites such as glycerol-3-phosphate. (**B**) During type 2 diabetes, increased phosphorylated p38 and JNK decreased AQP7 expression which may lead to decreased insulin secretion. (**C**) Metformin was capable to inhibit p38 and JNK phosphorylated and promoting AQP7 expression and insulin secretion. ATP: adenosine triphosphate; Ca^++^: calcium ions; DHAP: dihydroxyacetone phosphate GK: glycerol kinase; Glu: glucose; Gro: glycerol; Gro3P: glycerol-3-phosphate; JNK: c-Jun N-terminal kinase; K^+^: potassium ions; K_ATP_ channels: ATP-sensitive potassium channels; NAD^+^: nicotinamide adenine dinucleotide; NADH: reduced nicotinamide adenine dinucleotide; P: phosphate; p38: Mitogen-Activated Protein Kinase p38; Pyr: pyruvate VRAC: volume-regulated anion channel.

In vitro, extracellular isosmotic glycerol-induced sequential cell swelling, volume-regulated anion channel (VRAC) activation, membrane depolarization, calcium entry, and insulin secretion in both rat β-cells and pancreatic b-cell line BRIN-BD [291,297,298]. These effects are likely mediated by the entrance of glycerol and its subsequent metabolization [291]. Ex vivo, β-cells from *Aqp7* knockout mice displayed lower insulin secretion in response to D-glucose, extracellular hypotonicity or extracellular isosmotic addition of glycerol [292]. By allowing the passage of glycerol, AQP7 may regulate insulin secretion by acting at a distal downstream site in the insulin exocytosis pathway [292]. A recent analysis of AQP7 by cryo-electron microscopy and gas chromatography/mass spectrometry have suggested that the central pore formed by the tetrameric structure of AQP7 is permeable to glycerol-3-phosphate, while each individual pore formed by monomeric AQP7 is permeable to glycerol [290]. Furthermore, crystal structure revealed that the AQP7 dimer of tetramers of opposite lipidic layers interacts to form octamer [290]. It was hypothesized AQP7 octamer may serve as a junction protein and promote cell-cell adhesion between β-cells as well as in the rosette-like structures of b-cells around blood capillaries (also expressing AQP7) [290]. Hence, AQP7 could be considered a junctional protein. However, functional evidence for the role of AQP7 octamer in cell-cell adhesion is still lacking and further studies will be required to address this question. Therefore, AQP7 may facilitate intercellular movement and distribution of glycerol as well as other metabolites such as glycerol-3-phosphate. Considering adherens junction have been shown to be important for insulin secretion [299,300], AQP7 may therefore be involved in the control of insulin secretion through its function of glycerol and glycerol-3-phosphate channel and its feature to act as a junctional protein. Figure 5A recapitulates the role of AQP7 in β-cell physiology.

#### 6.2.2. Role of AQPs in Diseases Related to Altered Endocrine Pancreatic Secretion

Inflammation that has been involved in diabetes [301] can alter AQPs expression. Indeed, tumor necrosis factor α (TNF-α) reduced AQP7 and insulin secretion, but increased AQP12 levels, in rat pancreatic β-cell line RIN-m5F [302]. In the same cells, lipopolysaccharides raised both AQP7 and AQP12 levels but lowered insulin release [302]. Furthermore, in RIN-m5F cells incubated with (TNF-α) or lipopolysaccharides, overexpression and silencing of AQP7 or AQP12 showed the role of AQP7 in insulin release and of AQP12 in inflammation [302]. AQP8, expressed at the mitochondrial and plasma membranes of rat RIN-m5F β -cells, reduced cytokine-induced cell toxicity [303]. 

The human *Aqp7* gene is present in a chromosomal region that has been associated with type 2 diabetes [304] and metabolic syndrome [305]. In addition, single nucleotide polymorphisms in the *Aqp7* gene have been linked to obesity and/or type 2 diabetes in Caucasians [306,307] and with type 2 diabetes in the Chinese Han population [308]. Very recent data showed decreased *Aqp7* mRNA in islets from type 2 diabetic patients, a negative correlation between *Aqp7* mRNA expression in islets and body mass index, a strong association between a genetic variant of the *Aqp7* gene and random glucose and fasting glucose adjusted for body mass index [290]. Other variants of *Aqp7* gene were significantly associated with type 2 diabetes or glycated hemoglobin [290]. Altogether, data suggest that some genetic variants of *Aqp7* gene can be associated with metabolic features playing a role in type 2 diabetes. Still, the direct link between *Aqp7* gene genetic variants and protein dysfunction remains to be proven.

In obese patients with type 2 diabetes, sleeve gastrectomy induced diabetes remission possibly via an increase in glucagon-like peptide 1 (GLP-1) levels and a decrease in ghrelin levels [309,310]. Following sleeve gastrectomy in rats, it has been shown that increased GLP-1 levels and decreased ghrelin levels were responsible for AQP7 upregulation in b-cells which contributed to the improvement of insulin secretion and pancreatic steatosis [294]. *Aqp7* knockout mice displayed adult-onset obesity and hyperglycemia [293,295,311]. Altogether, these data suggest that dysregulated AQP7 expression is linked to dysregulated insulin secretion, thereof suggesting AQP7 as a potential therapeutic target for human obesity-associated type 2 diabetes. Interestingly, Metformin, a hypoglycemic drug used to treat diabetic patients, rescued AQP7 expression to induce insulin secretion in the rat in vitro and in vivo models of type 2 diabetes by suppressing the Mitogen-Activated Protein Kinase p38 and c-Jun N-terminal kinase (JNK) pathway [312]. Figure 5B,C recapitulate the role of AQP7 in β-cell during type 2 diabetes untreated or treated with Metformin.

While DNA methylation, an epigenetic modification, of the *Aqp7* gene promoter has been correlated with the decreased expression in human white adipose tissue and adiposity [313,314], further studies will be necessary to determine if such epigenetic modification can affect *Aqp7* gene expression and insulin secretion in β-cells.

Further studies are needed to pursue deciphering the role of AQPs in the pathogenesis of type 2 diabetes and associated inflammation.

## 7. Conclusions

Studies on the expression and role of AQPs throughout the gastrointestinal system have been highly instructive to provide useful insights into complex molecular mechanisms by which gastrointestinal exocrine and endocrine glands secrete and reabsorb water in concert with organic and inorganic compounds. In addition, important acquisitions have been made about the pathophysiological implication of AQPs in multiple gastrointestinal diseases accompanied by fluid imbalance. 

In salivary glands, AQP5 is the main AQP playing a key role in saliva secretion. In addition, deregulation of its expression may participate in the xerostomia manifesting in patients suffering from Sjögren’s syndrome, head and neck cancer treated with ionizing radiation therapy, or diabetes. Several therapeutic options have therefore been tested to modulate and/or restore the expression of AQP5 to alleviate xerostomia. In gastric glands, the role of AQP4 in gastric secretion remains a source of debate and a better understanding of its role in gastric cell volume maintenance will require further investigations. In gastric cancers, AQP3 and AQP5 may represent additional therapeutic targets for therapeutic intervention considering they have been shown to participate in the epithelial-mesenchymal transition of the gastric epithelium, to be regulated by miRNA and long coding RNA, and to represent interesting predictive prognostic biomarkers. So far, studies have solely investigated the expression of AQPs, but not their roles, in duodenal and intestinal secretory glands. So far, AQP5 and AQP1 have been hypothesized to participate in duodenal Brunner’s gland secretion, but additional studies will be worth to validate such a hypothesis. The role of AQP9 expression in intestinal goblet cells also remains to be studied further. In glands ensuring bile secretion, the expression and role of AQPs appear to be relatively specific to the different cell types involved including hepatocytes (AQP8 and AQP9), bile ducts (AQP1, AQP4), and gallbladder (AQP1 and AQP8). Deregulation of AQPs expression in cells involved in bile secretion has been linked to pathologies such as cholestasis and gallstone disease suggesting relevance as new therapeutic targets. Interestingly, hepatic gene transfer of human AQP1 has been reported to ameliorate the bile secretory failure in hepatocellular cholestasis by enhancing both biliary output and choleretic efficiency of key osmotic solutes such as bile salts and glutathione. Nevertheless, further studies will be valuable to deepen the current understanding of the complex mechanisms involved in bile secretion under physiological and pathological conditions. In the pancreas, the relative contribution of AQP1, AQP5, AQP8 and AQP12 to the exocrine secretion remains poorly understood. Some of these AQPs may play a role in cystic fibrosis and cancer and therefore, be considered interesting drug targets. Despite the detection of several AQPs in the endocrine pancreas, only AQP7 has been shown to date to participate in insulin secretion, and to undergo deregulation in type 2 diabetes that can be rescued by hypoglycemic drugs. Still, additional experiments will contribute to further precise the role of AQPs in exocrine and endocrine pancreatic secretions in healthy and pathological conditions.

Despite efforts made so far to decipher the regulation and precise function of AQPs in gastrointestinal glands, some questions remain open deserving additional studies. However, while studying the ultimate pathophysiological role of AQPs in several clinical disorders, novel pharmacological strategies might appear soon to treat gastrointestinal secretory/absorptive failures in which gastrointestinal glands epithelia are the target cell.

## Figures and Tables

**Table 1:** Location and roles of hepatobiliary aquaporins of functional relevance.

Hepatobiliary Section	AQP	Cellular Location and Species	Subcellular Location	Suggested Functional Involvement	References
Liver parenchyma	AQP3	Hep (h)	N.D.	Unclear	
	AQP7	Hep (h)	N.D.	Unclear	
	AQP8	Hep (r, m, h)	APM, SAV, IMM, SER	Canalicular bile secretion; cytoplasmic osmotic homeostasis; mitochondrial ammonia detoxification and ureagenesis; mitochondrial H_2_O_2_ release; cholesterol biosynthesis; regulation of metabolic signaling	[156,157,158,159]
	AQP9	Hep (r, m, h)	BLM	Glycerol uptake during starvation; lipid homeostasis; water movement from sinusoidal blood; catabolic urea secretion;	[156,160,161]
	AQP11	Hep (m)	RER	RER homeostasis; liver regeneration	
Intrahepatic bile ducts	AQP1	Chol (m, r, h)	APM, SAV, BLM	Secretion and absorption of ductal bile water	[41]
	AQP4	Chol (m, r)	BLM	Secretion and absorption of ductal bile water	[162]
Gallbladder	AQP1	EpC (m, h)	APM, BLM, SAV	Cystic bile absorption/secretion	[41,163,164,165]
	AQP8	EpC (m, h)	APM, SAV	Cystic bile absorption (?)	[163,164]
PS; PVP; BV	AQP1	EC (h)	APM, BLM	Bile formation and flow	[41]
Other hepatic cell types	AQP3	KC (h)	PM	Cell migration; proinflammatory cytokines secretion (?)	[156]
	AQP8	KC (r)	PM	Repopulation of Kupffer cells during liver regeneration (?)	[166]
	AQP3	SC (h)	PM	Adiponectin-mediated inhibition of SC cells activation	[167]
	AQP11	SC (r)	N.D.	Control of activated SC proliferation	[168]

AM, apical plasma membrane; BLM, basolateral plasma membrane; BV, blood vessels; EC: endothelial cells; EpC: epithelial cells; h: human; Hep: hepatocytes; IMM, inner mitochondrial membrane; KC: Kupffer cells; m: mouse; ND: not determined; PM, plasma membrane; PS: portal sinusoids; PVP, peribiliary vascular plexus; r; rat; RER, rough endoplasmic reticulum; SAV, subapical membrane vesicles; SC: stellate cells; SER, smooth endoplasmic reticulum.

## Data Availability

Not applicable.
